# Intra‐session and inter‐subject variability of 3D‐FID‐MRSI using single‐echo volumetric EPI navigators at 3T

**DOI:** 10.1002/mrm.28076

**Published:** 2019-11-13

**Authors:** Philipp Moser, Korbinian Eckstein, Lukas Hingerl, Michael Weber, Stanislav Motyka, Bernhard Strasser, Andre van der Kouwe, Simon Robinson, Siegfried Trattnig, Wolfgang Bogner

**Affiliations:** ^1^ High‐Field MR Center Department of Biomedical Imaging and Image‐guided Therapy Medical University of Vienna Vienna Austria; ^2^ Department of Biomedical Imaging and Image‐guided Therapy Medical University of Vienna Vienna Austria; ^3^ Athinoula A. Martinos Center for Biomedical Imaging, Department of Radiology Massachusetts General Hospital Harvard Medical School Boston Massachusetts; ^4^ Christian Doppler Laboratory for Clinical Molecular MR Imaging Vienna Austria

**Keywords:** concentric rings, dynamic functional magnetic resonance spectroscopic imaging, intra‐subject reproducibility, real time motion correction, reliability

## Abstract

**Purpose:**

In this study, we demonstrate the first combination of 3D FID proton MRSI and spatial encoding via concentric‐ring trajectories (CRTs) at 3T. FID‐MRSI has many benefits including high detection sensitivity, in particular for J‐coupled metabolites (e.g., glutamate/glutamine). This makes it highly attractive, not only for clinical, but also for, potentially, functional MRSI. However, this requires excellent reliability and temporal stability. We have, therefore, augmented this 3D‐FID‐MRSI sequence with single‐echo, imaging‐based volumetric navigators (se‐vNavs) for real‐time motion/shim‐correction (SHMOCO), which is 2× quicker than the original double‐echo navigators (de‐vNavs), hence allowing more efficient integration also in short‐TR sequences.

**Methods:**

The tracking accuracy (position and B_0_‐field) of our proposed se‐vNavs was compared to the original de‐vNavs in phantoms (rest and translation) and in vivo (voluntary head rotation). Finally, the intra‐session stability of a 5:40 min 3D‐FID‐MRSI scan was evaluated with SHMOCO and no correction (NOCO) in 5 resting subjects. Intra/inter‐subject coefficients of variation (CV) and intra‐class correlations (ICC) over the whole 3D volume and in selected regions of interest ROI were assessed.

**Results:**

Phantom and in vivo scans showed highly consistent tracking performance for se‐vNavs compared to the original de‐vNavs, but lower frequency drift. Up to ~30% better intra‐subject CVs were obtained for SHMOCO (*P* < 0.05), with values of 9.3/6.9/6.5/7.8% over the full VOI for Glx/tNAA/tCho/m‐Ins ratios to tCr. ICCs were good‐to‐high (91% for Glx/tCr in motor cortex), whereas the inter‐subject variability was ~11–19%.

**Conclusion:**

Real‐time motion/shim corrected 3D‐FID‐MRSI with time‐efficient CRT‐sampling at 3T allows reliable, high‐resolution metabolic imaging that is fast enough for clinical use and even, potentially, for functional MRSI.

## 1 INTRODUCTION

Proton MRSI (^1^H‐MRSI) allows the non‐invasive assessment of comprehensive neurochemical profiles in vivo^1^ and has been used to study many different neurologic, neuropsychiatric, and oncologic pathologies*.* 1 The simultaneous acquisition of multiple voxels allows for regional mapping of various neurochemicals at 3T, including NAA, Cr, Cho, myo‐inositol (m‐Ins), and glutamate + glutamine (Glx).^2^

At higher B_0_ of 7T or higher, FID‐based MRSI sequences with short acquisition delays have shown great potential for high‐resolution metabolic mapping.^3^, ^4^, ^5^ The benefits include negligible signal losses caused by T_2_‐relaxation or J‐modulation, low specific absorption rates, low chemical‐shift displacement errors, and reduced sensitivity to $B_{1}^{+}$errors. At 7T, FID‐MRSI sequences have been accelerated by non‐Cartesian k‐space sampling based on concentric‐ring trajectories (CRTs).^6^ For high spectral bandwidths, the self‐rewinding and constant‐angular velocity properties of CRTs render them more SNR‐efficient, faster, and less susceptible to gradient imperfections than other spatial‐spectral encoding approaches. Although 3T MRI scanners are more widely available, so far only a few studies have used CRTs,^7^, ^8^ only 2 studies used FID‐MRSI,^9^, ^10^ and no application has yet been reported for a combination of both at 3T. Besides being highly beneficial for clinical application, preliminary results^11^ also raise the hope that the efficient combination of FID‐MRSI and rapid CRT encoding could provide 3D mapping of glutamate with temporal and spatial resolutions high enough to observe stimuli‐induced changes via functional MRSI. However, this would require also excellent intra‐session stability, which is challenging to achieve.

One of the most common sources of artifacts in MRSI is subject motion. Motion artifacts are less obvious to recognize in MRSI compared to MR imaging,^12^ but can nevertheless severely degrade localization accuracy and spectral quality (e.g., line broadening, lipid contamination, and spectral peak splitting).^13^, ^14^ Rapid gradient switching within a heavy duty cycle sequence (e.g., CRTs, spirals, echo‐planar spectroscopic imaging [EPSI]) causes temporal B_0_ changes because of heating of the gradient coils and passive shims.^15^ A prospective (real‐time) correction that updates the position of the imaging volume, carrier frequency, and B_0_‐shim using volumetric, dual‐echo EPI navigators (vNavs) has already been demonstrated.^16^, ^17^, ^18^, ^19^ These vNavs are well‐suited to use in long‐TR sequences, where they can be deployed in the dead times between signal readout and the subsequent excitation^19^, ^20^ but prolong scan times of short‐TR sequences.

The aims of this study were 3‐fold: (1) to demonstrate that 3D‐FID‐MRSI accelerated by CRTs can provide high‐resolution metabolic maps in sufficiently short scan times at 3T; (2) to evaluate real‐time motion/shim correction based on shorter, single‐echo vNavs compared to previously proposed double‐echo vNavs; and (3) to report the intra‐session stability of real‐time motion/shim corrected 3D‐FID‐MRSI for major neurochemicals.

## 2 METHODS

### 2.1 Volunteers and hardware

This study was performed on a 3T Prisma MR scanner with a 64‐channel receive‐only head coil (all Siemens Healthineers) in 6 healthy volunteers (male/female, 4/2; age, 28.8 ± 5.4 y). Institutional Review Board approval and written, informed consent as well as a questionnaire to exclude abnormal medical conditions were obtained before the MR examinations.

### 2.2 Volumetric echo‐planar‐imaging navigators

**Figure 1:**
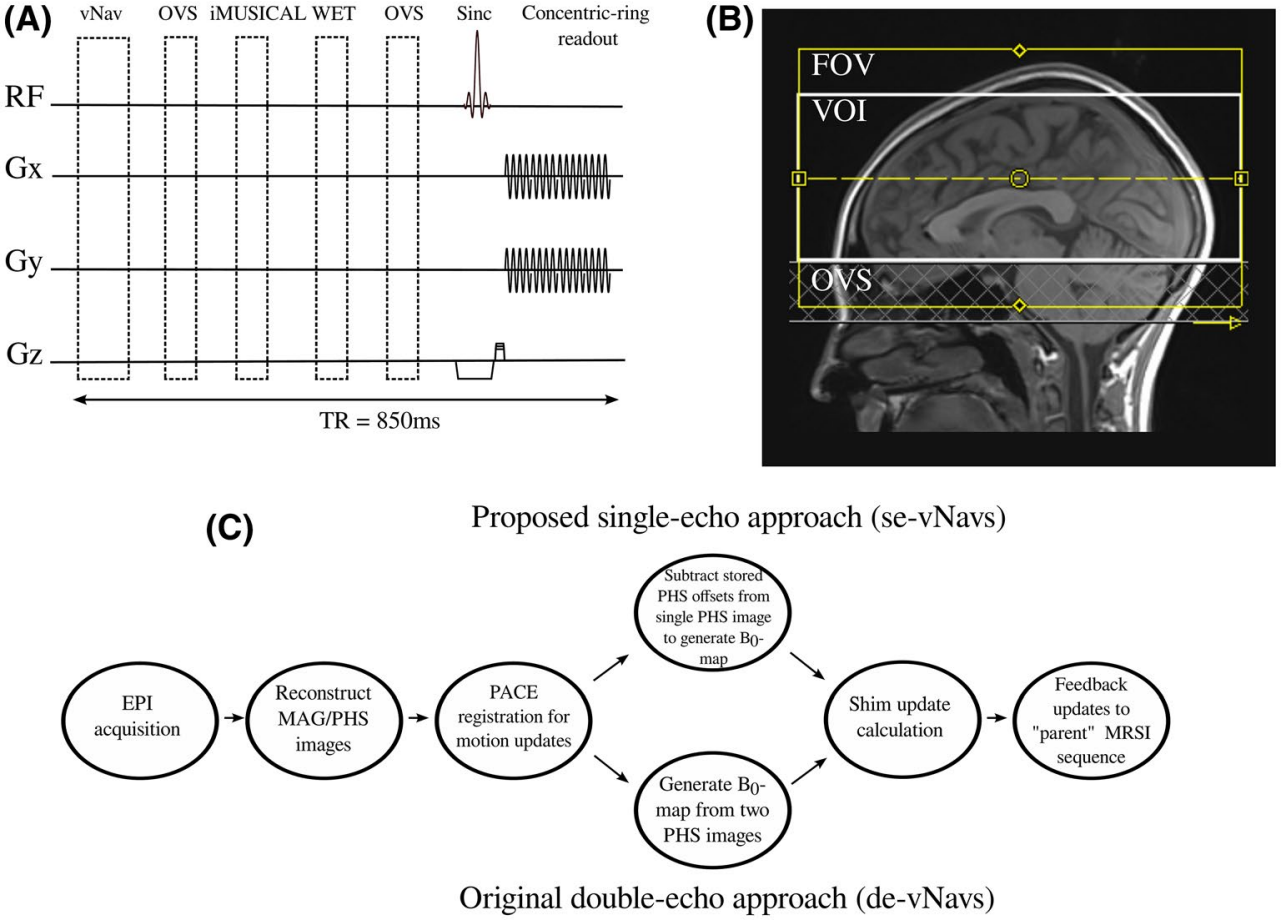
(A) Schematic diagram of the navigated 3D‐FID‐MRSI sequence: volumetric navigators (vNavs), iMUSICAL coil combination pre‐scan, water suppression enhanced through T_1_ effects (WET), outer‐volume‐suppression (OVS), and the 3D‐FID‐MRSI sequence with concentric‐ring readout (acquisition delay = 0.8 ms, TR = 850 ms). (B) Positioning of the MRSI volume including the placement of OVS slabs below the VOI. (C) Online reconstruction pipeline for our proposed single‐echo navigators and the original double‐echo navigators. The pipelines differ only by how the B_0_‐maps are created: for the single‐echo approach, the pre‐calculated phase offsets are subtracted from the single phase images, whereas for the double‐echo approach, B_0_‐maps are generated by subtracting the phase images from the 2 TEs

Originally, head pose and B_0_‐field changes were obtained in real‐time from dual‐echo, volumetric EPI navigators (de‐vNavs).^16^, ^19^ For this, each TR starts with a navigator acquisition followed by an immediate online EPI reconstruction to create B_0_‐maps and magnitude images (Figure 1A,C). Position changes are computed using PACE^21^ by co‐registering the magnitude images (TE_1_) of different time points.^16^, ^18^, ^19^ B_0_‐maps are generated per TR from phase images of both TEs. The total navigator block, including acquisition, reconstruction, update calculations, and feedback transmission of the current updates back to the MRSI sequence required ~760 ms.^19^

In contrast, our proposed single‐echo, volumetric EPI navigators (se‐vNavs) acquire only a single echo, resulting in only 1 magnitude/phase image per TR (Figure 1C). Calculating motion updates remains unchanged, because only the magnitude image from the first echo is used for calculating updates. Although B_0_‐maps are originally generated from the 2 TE phase images, we can reconstruct B_0_‐maps from the single phase images after subtracting pre‐stored, coil‐dependent phase offsets.^22^, ^23^ These are calculated using ASPIRE^24^ from a dual‐echo EPI reference pre‐scan whose parameters matched those of the se‐vNavs listed below, except for the dual‐echo TE_1_/TE_2_ of 7/14 ms (ASPIRE requires TE_2_ = 2 × TE_1_). The remaining processing pipeline (update calculation, feedback sending) is identical to the original pipeline (Figure 1C). Using our se‐vNavs, the total navigator block requires only ~360 ms.

The parameters for se‐vNavs were: TR = 17 ms; TE = 7 ms (de‐vNavs = TE_1_/TE_2_, 7/9.4 ms); matrix = 32 × 32; slices = 18; FOV = 256 × 256 × 144 mm^3^; bandwidth = 4734 Hz/pixel; flip angle = 4°; echo train length = 32; water excitation only; slice partial Fourier = 6/8.

### 2.3 Data acquisition

All sessions started with a 3D, T_1_‐weighted, MPRAGE sequence to position the MRSI volume (Figure 1B). B_0_‐map‐based, 1st and 2nd order B_0_‐shimming using standard Siemens routines was performed over the MRSI volume‐of‐interest (VOI).

The position of the navigator was identical to that of the phase offset reference scan covering the subject’s brain and was set using a rapid “setter” sequence^19^ before the MRSI sequence.

The 3D‐FID‐MRSI sequence (Figure 1A) used the following settings: TR= 850 ms; acquisition delay = 0.8 ms; flip angle = 70°; 600 µs sinc excitation pulse; B_1_ = 13.2 µT; VOI = 220 × 220 × 76 mm^3^; FOV = 220 × 220 × 126 mm^3^; in vivo matrix size = 50 × 50 × 21 (in phantoms = 32 × 32 × 21); complex spectral data points = 360; acquisition bandwidth = 1030 Hz; no temporal interleaving; acquisition window = 350 ms; averages = 1; maximum slew rate = 200 mT/m/ms; maximum gradient strength = 80 mT/m; spherical k‐space coverage; water suppression enhanced through T_1_‐effects (WET); outer‐volume saturation (OVS) band (30 mm thick) below VOI covering nasal cavity and skull base; TA = 5:40 min.

### 2.4 Data processing

All measured data were processed with an in‐house developed pipeline^25^ based on Bash (Free Software Foundation, Boston, MA) and MATLAB (The MathWorks, Natick, MA). The post‐processing pipeline included a modified Pipe‐Menon pre‐gridding density compensation,^26^ an off‐resonance correction,^27^ convolution gridding^28^ using a Kaiser‐Bessel kernel (width 3), coil‐wise L_2_‐lipid regularization^29^ and iMUSICAL coil combination.^30^ Detailed steps have been described previously.^6^, ^25^ LCModel 6.3^31^ was used to fit in vivo spectra in the spectral range of 1.8–4.2 ppm.

### 2.5 Part 1: tracking accuracy—se‐vNavs versus de‐vNavs

The accuracy and correlation of the position and B_0_‐field tracking (i.e., translation, rotation, frequency, and 1st‐order shims) were compared between se‐vNavs and de‐vNavs in phantom and in vivo measurements. To ensure comparable conditions for the se‐vNavs and de‐vNavs, the MRSI TR was set to 1120 ms to accommodate the more time‐consuming de‐vNavs. A head‐shaped gel phantom (6.3 L 1% agarose, 2 µmol/kg gadolinium, 0.1% NaCl, and 0.05% NaN_3_) was used.^32^ “Rest” and “push” phantom measurements were performed, where in the latter, the phantom was pushed once (4 mm into the scanner at 1:45 min). For the in vivo measurements, an MR‐experienced volunteer was trained and acoustically instructed to perform a head rotation of ~3° left–right (at 1:30 min), return to the initial position (at 2:00 min), perform a head rotation of ~3° left–right (at 3:00 min), and finally return to the initial position (at 3:30 min). A Styrofoam insert inside the coil providing the volunteer with a reference for the current head position ensured the reproducibility of the motion pattern.

### 2.6 Part 2: variability of 3D‐FID‐MRSI—no correction versus shim/motion‐correction

Five volunteers were scanned each within 1 session and without repositioning with a total of 8 3D‐FID‐MRSI scans: 4 no correction (NOCO) and 4 shim/motion‐correction (SHMOCO) in an interleaved fashion.

Metabolic concentration ratios (Glx/tCr, tNAA/tCr, tCho/tCr, and m‐Ins/tCr) were obtained on a voxel‐by‐voxel basis and voxels with poor spectral quality (i.e., CRLBs of tCr, tCh, tNAA, Glx, and m‐Ins >20%) were excluded from analysis. Mean SNR, FWHM, and CRLBs of Glx, tNAA, tCr, tCho, and m‐Ins were compared between SHMOCO and NOCO using paired t‐tests (*P* < 0.05 was considered statistically significant). Further, the intra‐subject (within volunteers) reproducibility and inter‐subject (between volunteers) variability were assessed by linear mixed effects models and are reported as coefficients of variation (CV). As a measure of method reliability, intra‐class correlation coefficients (ICC) were calculated by an absolute‐agreement, 2‐way, mixed‐effects model among the 4 SHMOCO and NOCO scans, respectively. Besides the entire VOI, specific brain regions were investigated in a region‐of‐interest (ROI)‐based analysis. ROIs with a volume of ~1 cm^3^ were manually placed in the upper part of the visual cortex (occipital lobe), in the motor cortex (frontal lobe), in the dorsolateral prefrontal cortex (DLPFC), and in the auditory cortex (temporal lobe). High‐resolution T_1_‐weighted images guided the consistent placing of ROIs in the same anatomic locations.

## 3 RESULTS

### 3.1 Part 1: tracking accuracy—se‐vNavs versus de‐vNavs

**Figure 2:**
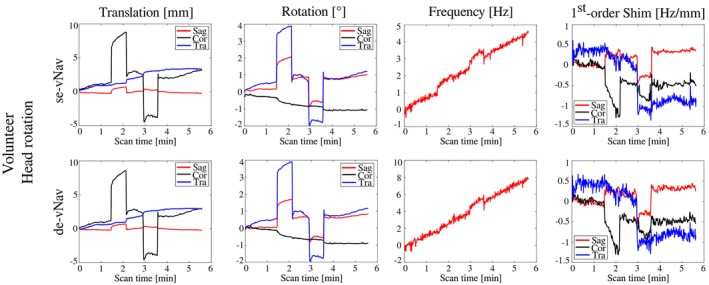
Comparison of the tracking performance of our proposed single‐echo navigators (se‐vNavs) and the original double‐echo navigators (de‐vNavs). Translation, rotation, frequency, and 1st‐order shim logs are shown for both navigator approaches. For the in vivo measurement, a MR‐trained volunteer was acoustically instructed to perform a predefined head rotation pattern

Figure 2 and Supporting Information Figure S1 show motion and B_0_‐field logs from the phantom and in vivo (volunteer 1) measurements. High similarities were found between the tracking of se‐vNavs and de‐vNavs. The mean differences of the total translation were 0.00 ± 0.01 mm, 0.01 ± 0.15 mm, and 0.01 ± 0.16 mm for the phantom “rest” and “push” as well as in vivo measurements, respectively (all R > 0.99, *P* < 0.001). The mean differences in 1st‐order shims (cor/sag/tra) were small and resulted in 0.01 ± 0.02/0.01 ± 0.03/0.05 ± 0.10 Hz/mm (all R > 0.97, *P* < 0.001) for the phantom in rest and 0.10 ± 0.06/0.06 ± 0.05/0.08 ± 0.05 Hz/mm (all R > 0.95, *P* < 0.001) for the in vivo scan. The frequency drift curves were similar in shape, but the total drifts for de‐vNavs were ~2.5‐fold (phantom) and ~1.7‐fold (in vivo) higher than for the se‐vNavs.

### 3.2 Part 2: variability of 3D‐FID‐MRSI—NOCO versus SHMOCO

**Figure 3:**
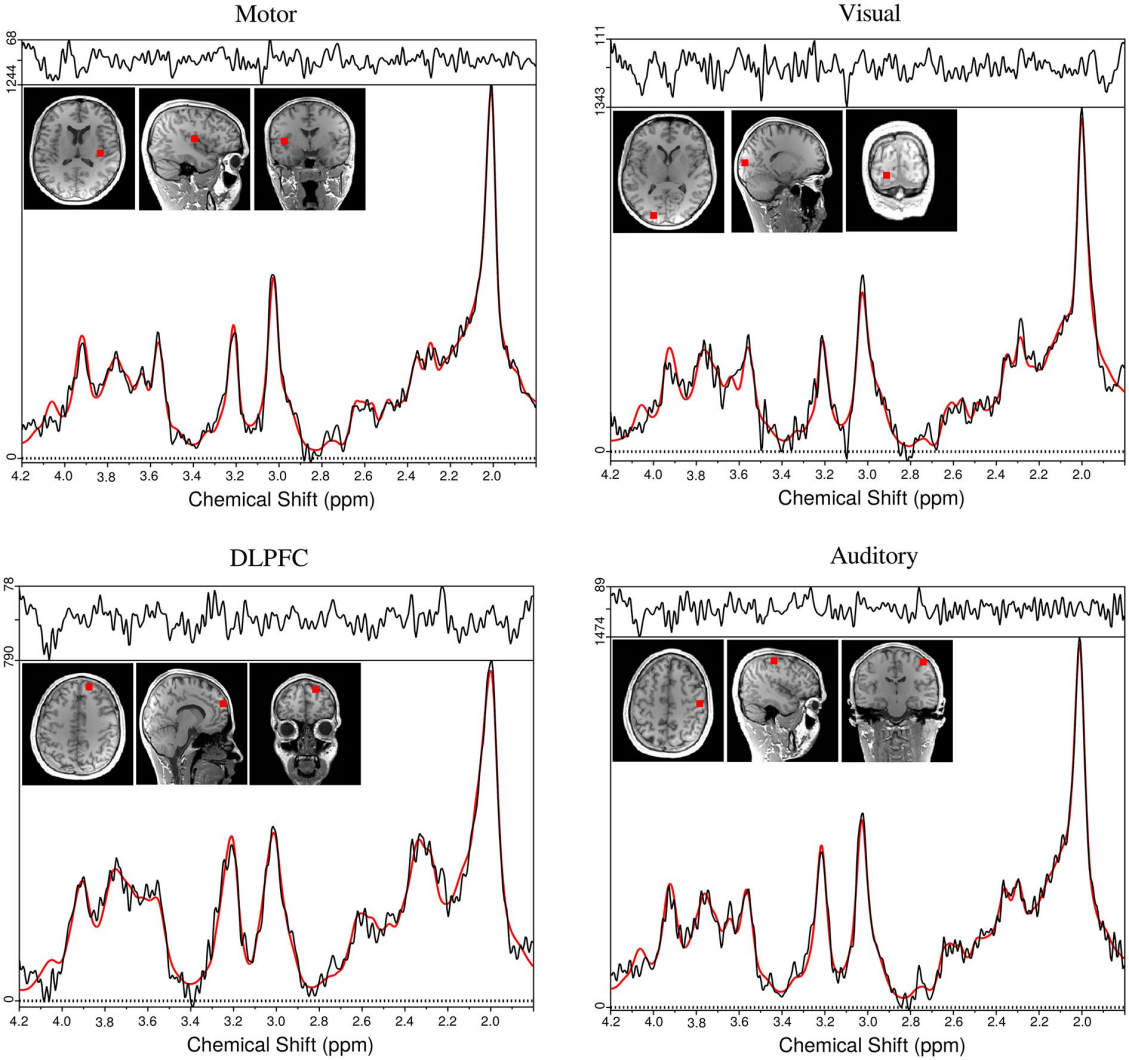
Representative spectra for volunteer 2 from 4 different ROIs (motor cortex, visual cortex, dorsolateral prefrontal cortex [DLPFC], and auditory cortex). For every ROI, the voxel position is marked on T_1_‐weighted images. The spectra are 1st‐order phase corrected for display

**Figure 4:**
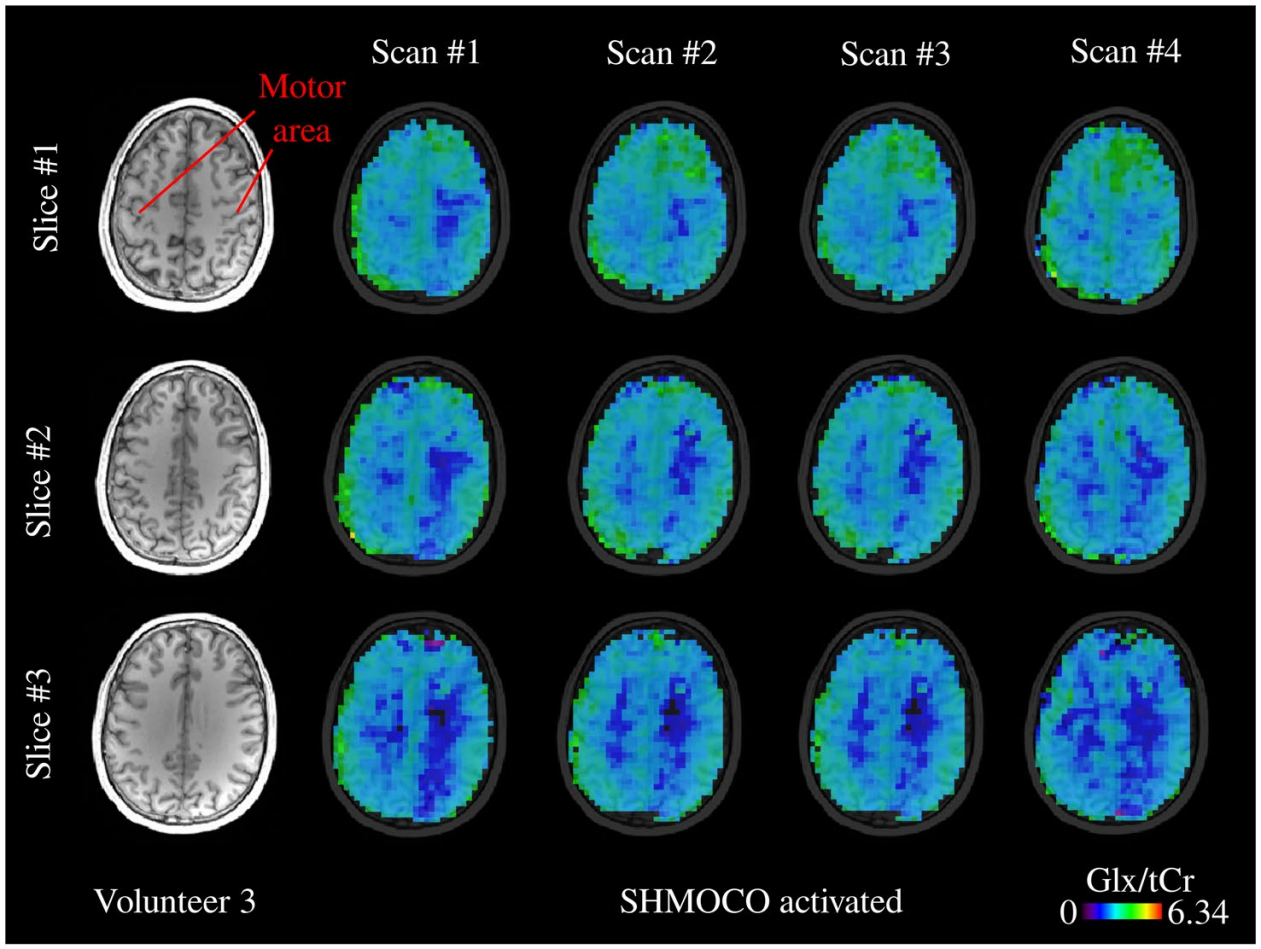
Metabolic ratio maps (Glx/tCr) for volunteer 3 depicted in 3 adjacent slices for the 4 scans with real‐time motion/shim correction turned on (SHMOCO); TR = 850 ms, acquisition delay = 0.8 ms, 50 × 50 × 21 matrix, nominal voxel size = 0.12 mL; TA = 5:40 min

Sample spectra for volunteer 2 from the above mentioned ROIs are shown in Figure 3. Figure 4 depicts the 4 Glx/tCr metabolic ratio maps with SHMOCO for volunteer 3 in 3 adjacent slices. Supporting Information Table S1 shows mean SNR, FWHM, and CRLBs of Glx, tNAA, tCr, tCho, and m‐Ins averaged over all 5 volunteers for the NOCO and SHMOCO measurements. Slightly improved results (higher SNR, lower FWHM, and lower CRLBs) were obtained for SHMOCO compared to NOCO, with only few values reaching statistical significance (e.g., SNR in DLPFC). Supporting Information Figure S2 shows scatter plots of the longitudinal measurements per subject for Glx/tCr and tNAA/tCr in the visual and motor cortex ROI.

The average maximum translations from the NOCO (0.9 ± 0.4 mm) and SHMOCO (1.2 ± 0.4 mm) scans were statistically non‐significant (*P* = 0.1).

Table 1 summarizes the means and SDs of the intra‐subject CVs obtained for the different metabolites ratios and brain regions averaged over all 5 volunteers. Intra‐subject CVs obtained with SHMOCO were all lower (i.e., better) compared to NOCO, with improvements of up to ~30%. Intra‐subject CVs of 9.3%, 6.9%, 6.5%, and 7.8% were obtained across the full VOI for Glx/tCr, tNAA/tCr, tCho/tCr, and m‐Ins/tCr with SHMOCO. For Glx/tCr, values of 9.8% and 8.2% were obtained in visual and motor cortex. Significant differences were found between NOCO and SHMOCO over the whole VOI (*P* < 0.001), but no moderation effect (*P* = 0.67) with the metabolites (i.e., no evidence that the difference between NOCO and SHMOCO) was more pronounced for certain metabolites. No moderation effect was also found with the ROIs and metabolites (i.e., no significant evidence that SHMOCO performed better for certain metabolites in certain ROIs) (*P* = 0.92). Paired t‐tests (NOCO vs. SHMOCO) for the individual ROIs and metabolites revealed significant differences in intra‐subject CVs for all metabolites in the full VOI, but only in 12 of 28 combinations of ROI and metabolite (e.g., Glx/tCr in the visual cortex) (Table 1). As measure of method reliability, ICCs are summarized in Supporting Information Table S2. Consistently better ICCs were found for SHMOCO compared to NOCO. Overall good (ICC >75%) to high (ICC >85%) measurement reliability was observed for SHMOCO with values of 85.1% and 91.1% for Glx/tCr in the visual and motor cortex, respectively. The inter‐subject variability for both SHMOCO and NOCO was generally (up to 2‐fold) higher than the intra‐subject variability and is summarized in Supporting Information Table S3.

**Table 1:** Mean and SDs of the intra‐subject CV obtained with SHMOCO and NOCO for different metabolic concentration ratios

Intra‐subject CV (%)	VOI	Visual	Motor_L	Motor_R	DLPFC_L	DLPFC_R	Auditory_L	Auditory_R
SHMOCO								
Glx/tCr	9.3 ± 1.0*	9.8 ± 1.1*	8.3 ± 0.7	8.2 ± 0.3	10.9 ± 1.9	10.3 ± 0.5	9.3 ± 0.3	10.1 ± 0.5
tNAA/tCr	6.9 ± 0.7*	8.1 ± 1.4	6.3 ± 0.9*	6.1 ± 0.4*	7.7 ± 1.1*	8.5 ± 1.0*	8.1 ± 1.5	8.8 ± 0.3
tCho/tCr	6.5 ± 1.0*	7.2 ± 1.0	6.0 ± 1.4	6.0 ± 0.8	7.0 ± 0.8*	7.2 ± 0.6*	6.9 ± 0.5*	7.6 ± 0.6*
m‐Ins/tCr	7.8 ± 1.4*	8.0 ± 1.3	6.7 ± 1.3	7.0 ± 1.5	8.7 ± 1.1*	9.0 ± 0.7*	8.2 ± 0.5*	9.1 ± 0.8
NOCO								
Glx/tCr	12.0 ± 1.6*	12.8 ± 1.2*	11.0 ± 1.8	11.3 ± 1.8	11.6 ± 1.8	12.2 ± 1.8	11.7 ± 1.3	12.5 ± 1.9
tNAA/tCr	9.6 ± 0.7*	10.3 ± 0.5	8.3 ± 0.6*	9.0 ± 0.7*	10.2 ± 0.8*	11.5 ± 0.8*	10.6 ± 0.4	10.9 ± 1.8
tCho/tCr	8.6 ± 0.7*	8.6 ± 1.3	7.6 ± 0.7	8.1 ± 1.1	9.3 ± 0.4*	10.0 ± 0.4*	9.8 ± 0.6*	9.5 ± 0.7*
m‐Ins/tCr	9.5 ± 1.4*	8.3 ± 0.9	8.3 ± 1.3	8.8 ± 1.2	10.7 ± 1.7*	11.1 ± 1.2*	10.8 ± 1.0*	10.8 ± 1.7

Abbreviations: CV, coefficients of variation; NOCO, no correction; SHMOCO, motion/shim‐correction.

* *P*‐value of < 0.05 was considered statistically significant.

## 4 DISCUSSION

We presented the first use of CRTs in a 3D‐FID‐MRSI sequence at 3T to generate high‐resolution metabolic maps in 5:40 min. The sequence was further equipped with a shorter, single‐echo volumetric EPI navigator for real‐time motion/shim correction. The performance of this novel navigator approach was compared to the previously published double‐echo navigator implementation, which had twice the duration. Finally, the temporal stability of the 3D‐FID‐MRSI sequence was assessed and the benefits of real‐time motion/shim correction were investigated in healthy volunteers.

Distinct anatomic contrasts could be observed (e.g., gray/white matter contrast in Glx or tCr)^9^ in the high‐resolution metabolic maps. The MRSI sequence was initially developed for 7T,^6^ but was adapted for 3T in this study and optimized by using a sub‐millisecond acquisition delay of 0.8 ms to maximize the signal and by shortening the water suppression module to reduce the TR.^33^ We acquired nominal voxel volumes of 0.12 mL in clinically highly feasible 5:40 min, which is significantly faster than most 3D‐MRSI reports from 3T. EPSI‐based studies report 0.31 mL voxels in 16.4 min^34^ or in 18 min with additional lipid inversion nulling,^35^ whereas non‐water‐suppressed multi‐band (3 slices) MRSI using density‐weighted CRTs has featured 0.25 mL voxels in 19.2 min.^36^ Overall, spectral quality in this study was good and allowed for reliable fitting of high‐concentrated metabolites (tNAA, tCr, tCho, m‐Ins, and Glx). However, the available SNR and spectral resolution at 3T limited the quantification of lower concentrated metabolites (e.g., glutathione, γ‐aminobutyric acid). Using a short acquisition delay of 0.8 ms made an improved optimization of lipid contamination via L_2_‐lipid regularization and macromolecular signals^37^ necessary because of their fast T_2_‐relaxation.

The potential for correction of motion and scanner‐instability related artifacts using vNavs has been shown in a broad range of MR techniques including anatomic imaging,^38^ diffusion,^39^ chemical exchange saturation transfer,^40^ and edited/non‐edited MRS/MRSI.^16^, ^20^, ^41^ All of these methods provide sufficient sequence dead time to incorporate vNavs without prolonging scan times. Because of their shortened acquisition time, our proposed se‐vNavs can also be readily integrated in MR sequences with little dead time, such as FID‐MRSI. Similar to Dymerska et al.,^22^ who generated B_0_‐maps for the dynamic correction of single‐echo EPI at 7T, we also used a dual‐echo reference scan for the calculation of phase offsets, which have been shown to be stable during long measurements and for large head motions. However, instead of a gradient echo pre‐scan, we used the same EPI readout as for the vNavs to minimize geometrical misalignments. Phantom and in vivo tests showed a high agreement in the position and B_0_‐field tracking between our se‐vNavs and the original de‐vNavs. An additional advantage of se‐vNavs can be observed in the frequency drift curves, where the drift is reduced by a factor of ~2 compared to de‐vNavs because of less gradient‐intensive EPI readout. Using SHMOCO led to moderately higher SNR and lower CRLBs compared to NOCO, whereas the FWHMs were little affected. However, the intra‐session stability of metabolic ratio maps was significantly improved, which could potentially have important implications for functional MRSI (fMRSI).

Functional MRS studies have reported changes in glutamate^42^ range from only subtle increases of 2–4% after visual or motor stimuli to more pronounced changes (up to 22%) after pain stimuli. Although fMRSI has not yet been conducted so far, this report contributes to paving the way to fMRSI by assessing the stability of 3D‐FID‐MRSI that makes it possible to judge if small metabolite changes can actually be resolved on a single‐subject basis or on a group level. To date, most reproducibility MRSI studies have focused on longitudinal measurements (repeated scans across 1 or several days with repositioning). Zhang et al^35^ have reported mean intra‐subject CVs in a longitudinal study (3 separate sessions) of 7.6% for metabolites relative to Cr, while Maudsley et al^43^ obtained median intra‐subject CVs of 6.2%, 7.2%, and 9.7%, for NAA, Cr, and Cho, respectively, from 5 separate sessions. Only a small number of studies have investigated the intra‐session stability of 3D‐MRSI to date. Comparisons between these inter‐ and intra‐session CVs are not straight‐forward, but generally lower CVs are expected without repositioning. Ding et al^44^ have assessed the intra‐session reproducibility and found metabolite CVs of 12.8%, 19.3%, 14.5%, 30.6%, and 30.6% for NAA, tCho, tCr, Glx, and m‐Ins, respectively, whereas Bian et al^45^ have reported an intra‐session CV of the metabolite peak height of 12.4%. The intra‐subject CVs obtained in this study (Glx/tCr: 9.3%, tNAA/tCr: 6.9%, tCho/tCr: 6.5%, m‐Ins/tCr: 7.8%) were significantly lower than those obtained in the above‐mentioned intra‐session reports. Further improvements in intra‐subject CVs compared to 8–10% in Glx/tCr as observed here can be expected with higher SNR and spectral resolution at ≥7T,^9^, ^46^ potentially enabling functional MRSI studies. Our results also confirm that the use of SHMOCO gives overall significantly better intra‐subject CVs than NOCO, which mainly reflects SHMOCO’s ability to correct for temporal frequency drifts and involuntary subject movements. Method reliabilities measured as ICCs were good to high and improved for SHMOCO compared to NOCO. The inter‐subject variability driven by differences in positioning, shim settings, and subject condition was generally higher than the intra‐session reproducibility, which is in accordance with literature.^47^

### 4.1 Limitations

As an explorative proof‐of‐principle study, the number of volunteers was limited, thereby limiting the statistical power of the analysis. Nevertheless, a clear trend toward improved results when using SHMOCO compared to NOCO has been observed. Concerning motion correction, comparisons of methods without a ground truth (i.e., an ideally non‐moving subject) always need to be interpreted with caution.

For a real dynamic/functional MRSI experiment, instead of performing repeated MRSI scans, it would be more suitable to run a single scan with multiple measurements, which has not yet been implemented nor possible because of the amount of raw data generated.

The duration of se‐vNavs is still fairly long for integration into some MR sequences. Further acceleration could be achieved by k‐space undersampling such as shown for FatNavs.^48^

## 5 CONCLUSIONS

3D‐FID‐MRSI at 3T allows for reliable, high‐resolution metabolic imaging in clinically attractive scan times. Implementing accelerated, single‐echo, volumetric, imaging‐based navigators reduced the susceptibility to motion and B_0_‐instabilities and increased the spectral quality, while providing similar tracking accuracy as previously published double‐echo navigators. Using real‐time motion/shim correction improved the intra‐session stability of 3D‐FID‐MRSI and makes it a potential basis for 3D‐fMRSI.

## Supporting information


**FIGURE S1** Comparison of the tracking performance of our proposed single‐echo navigators (se‐vNavs) and the original double‐echo navigators (de‐vNavs). Translation, rotation, frequency, and 1st‐order shim logs are shown for both navigator approaches. Phantom measurements were performed for “rest” and “push” conditions, where in the latter the phantom was manually pushed 4 mm into the scanner after 1:45 min
**FIGURE S2** Scatter plots of the longitudinal measurements for all 5 subjects in the visual and motor cortex ROI. The 4 time points from the SHMOCO scans are depicted for Glx/tCr and tNAA/tCr (i.e., the mean concentrations within the aforementioned ROIs obtained from the 4 SHMOCO scans)
**TABLE S1** Mean and SDs of SNR, FWHM, and metabolic CRLB values obtained with (SHMOCO) and without (NOCO) shim/motion correction for different metabolic concentration ratios. *P‐value of <0.05 was considered statistically significant
**TABLE S2** Method reliability measured as intra‐class correlation coefficients (ICCs). All values are given in percent and include mean ICC and lower and upper bounds (in brackets) with alpha level of significance of 0.5. A clear trend toward higher method reliability was observed for SHMOCO compared to NOCO
**TABLE S3** Means and SDs of the inter‐subject variability expressed as coefficients of variation (CV) for SHMOCO and NOCO. Slightly better (i.e., lower) values were obtained for SHMOCO
