# Supplementary material for: Intra‐session and inter‐subject variability of 3D‐FID‐MRSI using single‐echo volumetric EPI navigators at 3T
Source: Magn Reson Med. 2019 Nov 13;83(6):1920–9. doi: 10.1002/mrm.28076 (PMC7065144; doi:10.1002/mrm.28076)
Supplement: Supplementary file 1 — FIGURE S1 Comparison of the tracking performance of our proposed single‐echo navigators (se‐vNavs) and the original double‐echo navigators (de‐vNavs). Translation, rotation, frequency, and 1st‐order shim logs are shown for both navigator approaches. Phantom measurements were performed for “rest” and “push” conditions, where in the latter the phantom was manually pushed 4 mm into the scanner after 1:45 min FIGURE S2 Scatter plots of the longitudinal measurements for all 5 subjects in the visual and motor cortex ROI. The 4 time points from the SHMOCO scans are depicted for Glx/tCr and tNAA/tCr (i.e., the mean concentrations within the aforementioned ROIs obtained from the 4 SHMOCO scans) TABLE S1 Mean and SDs of SNR, FWHM, and metabolic CRLB values obtained with (SHMOCO) and without (NOCO) shim/motion correction for different metabolic concentration ratios. *P‐value of <0.05 was considered statistically significant TABLE S2 Method reliability measured as intra‐class correlation coefficients (ICCs). All values are given in percent and include mean ICC and lower and upper bounds (in brackets) with alpha level of significance of 0.5. A clear trend toward higher method reliability was observed for SHMOCO compared to NOCO TABLE S3 Means and SDs of the inter‐subject variability expressed as coefficients of variation (CV) for SHMOCO and NOCO. Slightly better (i.e., lower) values were obtained for SHMOCO [file MRM-83-1920-s001.docx]

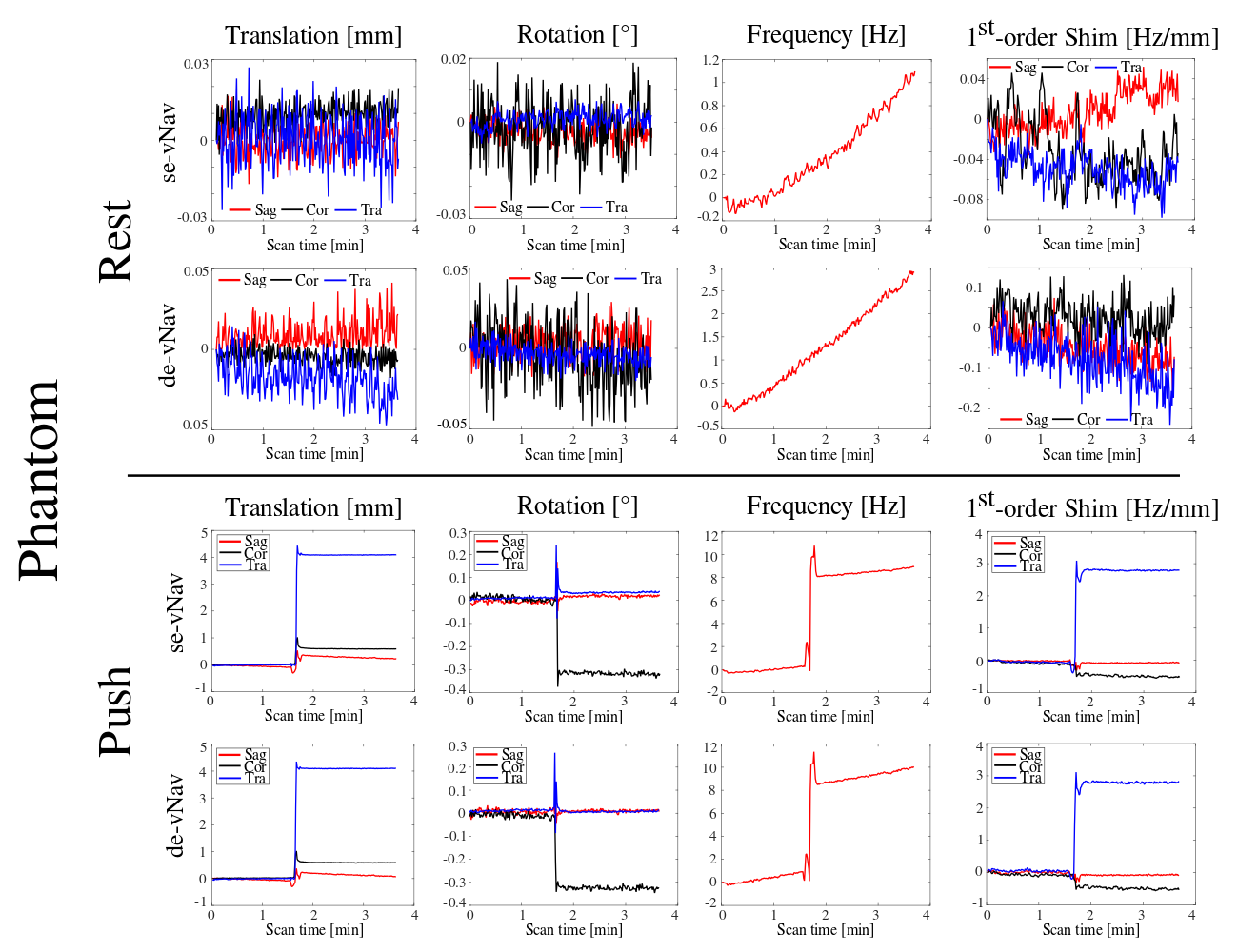


Supporting Information Figure S1: Comparison of the tracking performance of our proposed single-echo navigators (se-vNavs) and the original double-echo navigators (de-vNavs). Translation, rotation, frequency, and 1^st^-order shim logs are shown for both navigator approaches. Phantom measurements were performed for “rest” and “push” conditions, where in the latter the phantom was manually pushed 4mm into the scanner after 1:45min.

| **SHMOCO** | | | | | | |
| --- | --- | --- | --- | --- | --- | --- |
|  | VOI | Visual | Motor | | DLPFC | Auditory |
| SNR [a.u.] | 16.5±5.3 | 16.3±4.7 | 17.8±5.0 | | 15.5±5.0* | 12.0±4.1 |
| FWHM [Hz] | 8.2±2.8 | 7.3±1.8 | 7.2±2.1 | | 10.3±2.9 | 8.6±2.7 |
| CRLB Glx [%] | 8.8±2.8 | 9.3±2.8 | 8.7±2.7 | | 8.5±2.7 | 9.2±3.0 |
| CRLB tNAA [%] | 4.1±1.3 | 4.3±1.0 | 4.0±1.0 | | 4.3±1.6 | 4.7±1.5 |
| CRLB tCr [%] | 5.9±1.9 | 6.3±1.7 | 5.8±1.7 | | 5.7±2.0 | 6.4±1.9 |
| CRLB tCho[%] | 6.5±1.9 | 5.9±1.3* | 6.1±1.7 | | 6.7±2.1 | 7.4±2.3 |
| CRLB m-Ins [%] | 6.1±1.7 | 5.9±1.3 | 5.7±1.3 | | 6.3±1.9 | 7.0±1.9 |
| **NOCO** | | | | | | |
|  | VOI | Visual | Motor | DLPFC | | Auditory |
| SNR [a.u.] | 15.9±5.3 | 15.3±4.7 | 17.8±5.3 | 13.8±4.0* | | 12.0±4.0 |
| FWHM [Hz] | 8.4±2.5 | 8.5±2.3 | 7.3±1.9 | 9.8±2.7 | | 8.9±2.7 |
| CRLB Glx [%] | 8.8±2.8 | 9.6±2.9 | 8.5±2.7 | 9.2±2.7 | | 9.3±3.0 |
| CRLB tNAA [%] | 4.2±1.5 | 4.0±1.4 | 3.8±1.3 | 4.4±1.8 | | 4.7±1.7 |
| CRLB tCr [%] | 6.0±2.2 | 7.1±2.6 | 5.8±2.0 | 6.0±2.3 | | 6.2±2.0 |
| CRLB tCho [%] | 7.1±2.4 | 7.5±2.1* | 6.8±2.0 | 8.2±2.6 | | 8.3±2.6 |
| CRLB m-Ins [%] | 5.9±1.8 | 6.6±1.9 | 5.5±1.5 | 6.5±2.0 | | 6.7±2.0 |

Supporting Information Table S1: Mean and standard deviations of SNR, FWHM and metabolic CRLB values obtained with (SHMOCO) and without (NOCO) shim/motion correction for different metabolic concentration ratios. A p-value of < 0.05 was considered statistically significant and is marked with a single asterisk.


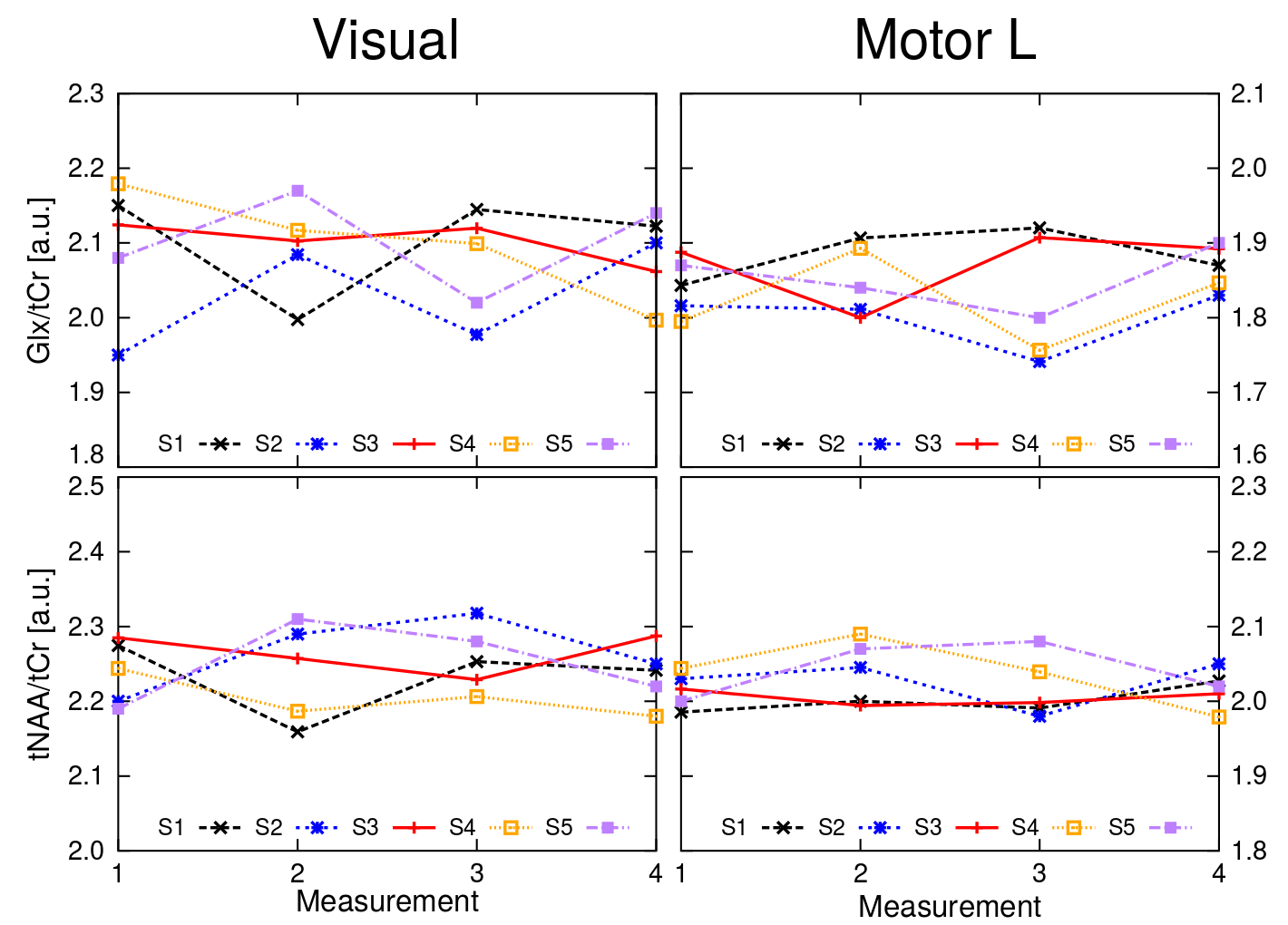


Supporting Information Figure S2: Scatter plots of the longitudinal measurements for all five subjects in the visual and motor cortex ROI. The four time points from the SHMOCO scans are depicted for Glx/tCr and tNAA/tCr, i.e., the mean concentrations within the aforementioned ROIs obtained from the four SHMOCO scans.

| **SHMOCO – Intraclass correlation coefficient (ICC) [%]** | | | | | |
| --- | --- | --- | --- | --- | --- |
|  | VOI | Visual | Motor_L | DLPFC_L | Auditory_L |
| Glx/tCr | 79.6 (79.3/79.8) | 85.1 (84.8/85.4) | 91.1 (91.0/91.2) | 76.7 (76.1/77.3) | 84.3 (84.0/84.6) |
| tNAA/tCr | 77.2 (76.9/77.5) | 80.7 (80.4/81.1) | 83.5 (83.3/83.8) | 81.9 (81.5/82.3) | 72.5 (71.9/73.0) |
| tCho/tCr | 81.6 (81.5/81.8) | 95.2 (95.1/95.3) | 96.2 (96.2/96.3) | 85.4 (85.0/85.7) | 85.0 (84.7/85.3) |
| m-Ins/tCr | 76.8 (76.7/77.0) | 85.8 (85.5/86.0) | 92.6 (92.5/92.7) | 86.1 (85.8/86.4) | 77.9 (77.6/78.2) |
| **NOCO – Intraclass correlation coefficient (ICC) [%]** | | | | | |
|  | VOI | Visual | Motor_L | DLPFC_L | Auditory_L |
| Glx/tCr | 70.0 (69.8/70.3) | 81.1 (80.6/81.6) | 87.4 (87.2/87.6) | 68.6 (67.6/69.6) | 70.9 (70.4/71.3) |
| tNAA/tCr | 78.3 (78.0/78.5) | 75.8 (75.1/76.4) | 82.5 (82.2/82.8) | 77.1 (76.3/77.8) | 60.8 (60.2/61.4) |
| tCho/tCr | 80.6 (80.4/80.8) | 92.2 (92.0/92.4) | 93.1 (93.0/93.2) | 78.6 (77.9/79.3) | 68.7 (68.3/69.2) |
| m-Ins/tCr | 70.1 (69.7/70.4) | 80.3 (69.5/71.1) | 80.5 (80.1/80.8) | 64.9 (53.4/56.3) | 66.3 (65.7/67.0) |

Supporting Information Table S2: Method reliability measured as Intraclass correlation coefficients (ICCs). All values are given in percent and include mean ICC and lower and upper bounds (in brackets) with alpha level of significance of 0.5. A clear trend towards higher method reliability was observed for SHMOCO compared to NOCO.

| **SHMOCO – Inter-subject coefficients of variation (CV) [%]** | | | | | |
| --- | --- | --- | --- | --- | --- |
|  | VOI | Visual | Motor_L | DLPFC_L | Auditory_L |
| Glx/tCr | 18.1 ± 11.4 | 17.7 ± 8.7 | 16.6 ± 9.7 | 19.4 ± 12.5 | 18.4 ± 11.3 |
| tNAA/tCr | 13.3 ± 9.5 | 14.1 ± 8.3 | 10.2 ± 5.8 | 13.9 ± 10.0 | 13.8 ± 9.3 |
| tCho/tCr | 12.3 ± 8.3 | 11.5 ± 7.4 | 11.3 ± 7.4 | 12.4 ± 8.0 | 12.6 ± 8.9 |
| m-Ins/tCr | 15.0 ± 9.9 | 10.7 ± 6.3 | 14.9 ± 8.2 | 16.8 ± 11.3 | 13.9 ± 9.1 |
| **NOCO – Inter-subject coefficients of variation (CV) [%]** | | | | | |
|  | VOI | Visual | Motor_L | DLPFC_L | Auditory_L |
| Glx/tCr | 19.6 ± 11.9 | 18.1 ± 10.6 | 16.7 ± 9.5 | 21.8 ± 12.5 | 18.4 ± 12.1 |
| tNAA/tCr | 14.0 ± 9.1 | 15.9 ± 9.7 | 12.6 ± 7.7 | 15.6 ± 10.4 | 14.8 ± 10.2 |
| tCho/tCr | 17.1 ± 9.8 | 17.1 ± 9.1 | 13.3 ± 7.0 | 20.4 ± 10.6 | 16.1 ± 11.0 |
| m-Ins/tCr | 14.9 ± 10.1 | 13.4 ± 8.5 | 12.8 ± 8.1 | 17.6 ± 12.3 | 15.7 ± 11.3 |

Supporting Information Table S3: Means and standard deviations of the inter-subject variability expressed as coefficients of variation (CV) for SHMOCO and NOCO. Slightly better (i.e., lower) values were obtained for SHMOCO.
